# SHMT1 and SHMT2 Are Functionally Redundant in Nuclear *De novo* Thymidylate Biosynthesis

**DOI:** 10.1371/journal.pone.0005839

**Published:** 2009-06-09

**Authors:** Donald D. Anderson, Patrick J. Stover

**Affiliations:** 1 Graduate Field of Biochemistry, Molecular and Cellular Biology, Cornell University, Ithaca, New York, United States of America; 2 Division of Nutritional Sciences, Cornell University, Ithaca, New York, United States of America; National Institutes of Health (NIH)/National Institute of Environmental Health Sciences (NIEHS), United States of America

## Abstract

The three enzymes that constitute the *de novo* thymidylate synthesis pathway in mammals, cytoplasmic serine hydroxymethyltransferase (SHMT1), thymidylate synthase (TYMS) and dihydrofolate reductase (DHFR) undergo sumoylation and nuclear import during S-phase. In this study, we demonstrate that purified intact mouse liver nuclei convert dUMP to dTMP in the presence of NADPH and serine. Neither nuclear extracts nor intact nuclei exposed to aminomethylphosphonate, a SHMT inhibitor, exhibit thymidylate synthesis activity. Nuclei isolated from *Shmt1^−/−^* mouse livers retained 25% of thymidylate synthesis activity exhibited by nuclei isolated from wild type mice. This residual activity was due to the presence of a cytoplasmic/nuclear isozyme of SHMT encoded by *Shmt2*. *Shmt2* is shown to encode two transcripts, one which encodes a protein that localizes exclusively to the mitochondria (SHMT2), and a second transcript that lacks exon 1 and encodes a protein that localizes to the cytoplasm and nucleus during S-phase (SHMT2α). The ability of *Shmt2* to encode a cytoplasmic isozyme of SHMT may account for the viability of *Shmt1^−/−^* mice and provide redundancy that permitted the expansion of the human *SHMT1* L474F polymorphism that impairs SHMT1 sumoylation and nuclear translocation.

## Introduction

**Figure 1:**
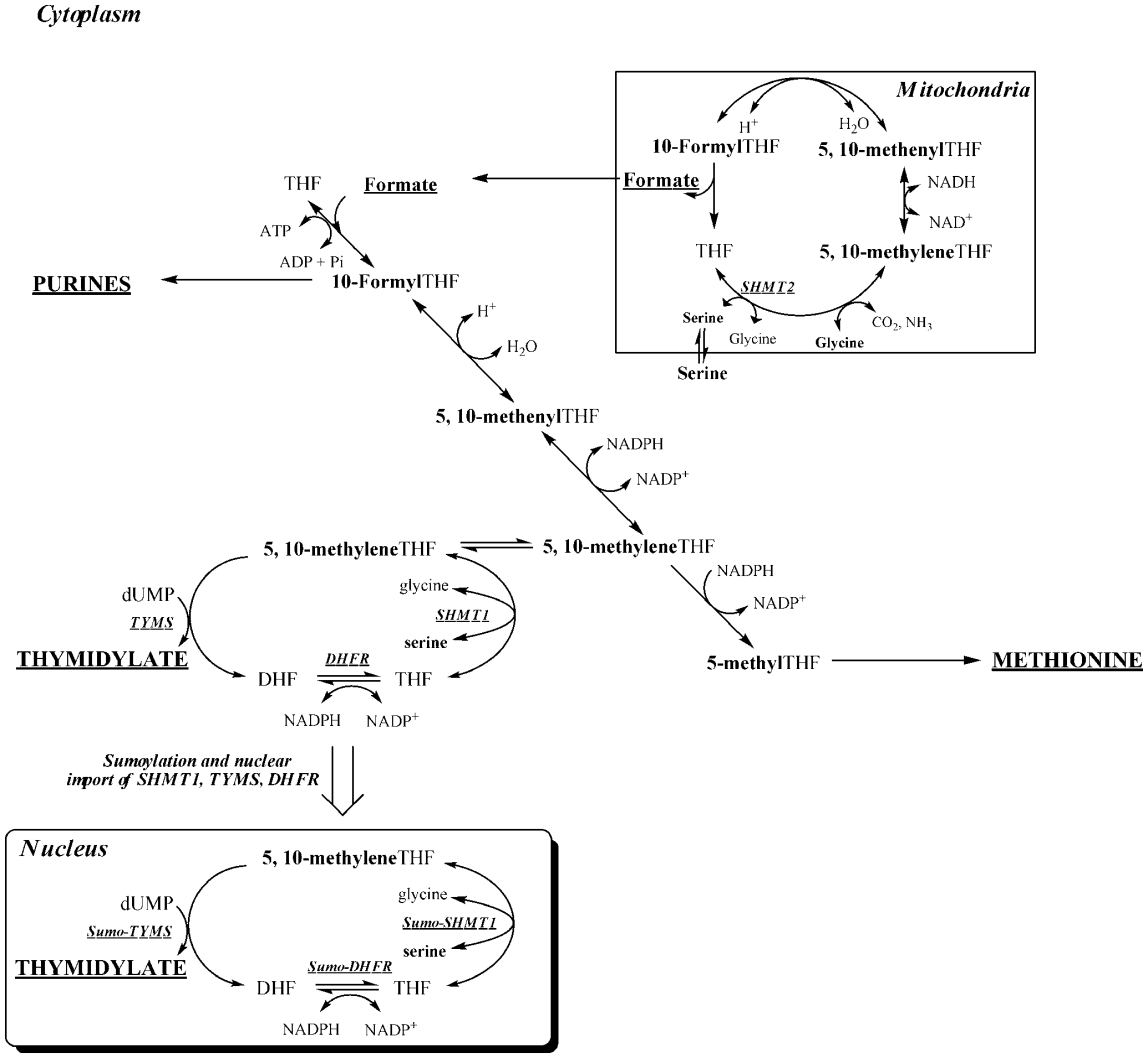
Compartmentation of folate-mediated one-carbon metabolism in the cytoplasm, mitochondria and nucleus. One-carbon metabolism in the cytoplasm is required for the *de novo* synthesis of purines and thymidylate, and for the remethylation of homocysteine to methionine. One-carbon metabolism in mitochondria generates one-carbon units for cytoplasmic one-carbon metabolism by generating formate from serine and glycine. One-carbon metabolism in the nucleus synthesizes dTMP from dUMP and serine. SHMT2, mitochondrial serine hydroxymethyltransferase; SHMT1, cytoplasmic serine hydroxymethyltransferase; TYMS, thymidylate synthase; DHFR, dihydrofolate reductase; THF, tetrahydrofolate.

Tetrahydrofolate (THF) is a metabolic cofactor that carries and activates single carbons for the synthesis of nucleotides and methionine [1]. Folate-mediated one-carbon metabolism is compartmentalized in the mitochondria and cytoplasm of eukaryotic cells (Figure 1). In the cytoplasm, this metabolic network is required for the biosynthesis of purines, thymidylate, and the remethylation of homocysteine to form methionine. Serine is a major source of one-carbon units for this network through its reversible and tetrahydrofolate-dependent conversion to glycine and methyleneTHF catalyzed by serine hydroxymethyltransferase (SHMT). There are cytoplasmic and mitochondrial SHMT isozymes. *SHMT1* encodes the cytoplasmic isozyme (SHMT1) and *SHMT2* encodes the mitochondrial isozyme (SHMT2) [2], [3], [4]. Mitochondrial one-carbon metabolism generates one-carbons from serine through the activity of SHMT2, and the one-carbon is oxidized and exported to the cytoplasm as formate, supporting cytoplasmic one-carbon metabolism [5]. The SHMT1 enzyme generates methyleneTHF for thymidylate and methionine biosynthesis, but isotope tracer studies indicate that SHMT1 preferentially partitions methyleneTHF to thymidylate biosynthesis [6].

The *de novo* thymidylate biosynthesis pathway requires three enzymes: thymidylate synthase (TYMS), dihydrofolate reductase (DHFR), and SHMT1. MethyleneTHF generated by SHMT is the one-carbon donor for the TYMS catalyzed conversion of dUMP to dTMP generating dihydrofolate (DHF). DHFR catalyzes the NADPH-dependent reduction of DHF to regenerate THF for subsequent cycles of *de novo* thymidylate synthesis. Recently, the enzymes that constitute the thymidylate synthesis cycle were shown to undergo post-translational modification by the small ubiquitin-like modifier (SUMO) and nuclear translocation during S and G2/M phases [7], [8]. Although the synthesis of thymidylate in the nucleus has never been demonstrated, others have found folate cofactors present in liver nuclei [9], and multi-enzyme complexes containing ribonucleotide reductase and thymidylate synthase have been isolated from nuclear extracts [10]. In this study, intact nuclei are shown to catalyze the formation of dTMP from dUMP, which accounts for the results of stable isotope studies that indicate SHMT preferentially partitions methyleneTHF to thymidylate biosynthesis. Furthermore, both *Shmt1* and *Shmt2* are shown to contribute to nuclear *de novo* thymidylate biosynthesis.

## Results

**Figure 2:**
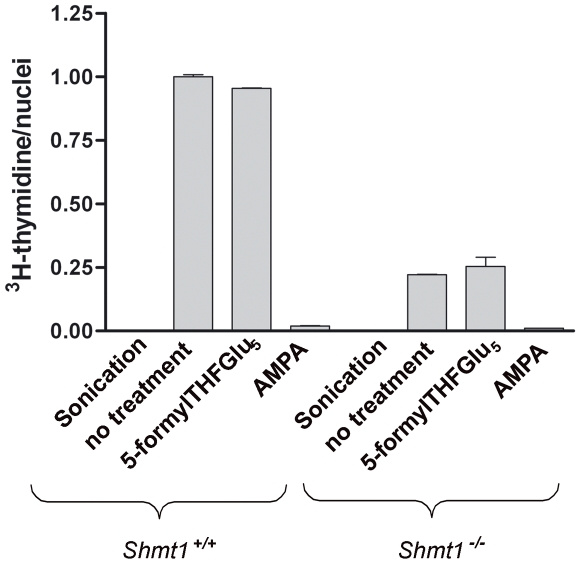
Thymidylate biosynthesis occurs in purified nuclei. Liver nuclei were isolated from *SHMT1^+/+^* and *SHMT1^−/−^* mice and capacity to convert dUMP and [2,3-^3^H]-L-serine to [^3^H]dTMP was determined in reactions that contained: 1) sonicated nuclei; 2) intact nuclei; 3) intact nuclei with 200 µM 5-CHOTHFGlu_5_; and 4) intact nuclei with 100 mM aminomethyl phosphonate (AMPA). *De novo* thymidylate biosynthesis activities were normalized to activity generated from *SHMT1^+/+^* intact nuclei which was given an arbitrary value of 1.0. Reactions containing sonicated nuclei contained no activity. All reactions were performed in duplicate and the experiment repeated twice. Variation is expressed as the standard deviation.

*Shmt1* and *Shmt2* contribute to nuclear dTMP biosynthesis. The ability of purified nuclei to catalyze the formation of tritiated dTMP from unlabeled dUMP, NADPH and [2,3-^3^H]-L-serine *in vitro* was investigated (Figure 2). *Intact* nuclei isolated from the livers of wild type mice were capable of generating tritiated dTMP, demonstrating that folate-dependent nuclear dTMP synthesis occurs in liver. The addition of the SHMT inhibitor and amino acid analog, aminomethylphosphonate, to the reaction mixture inhibited dTMP synthesis by greater than 95%, demonstrating the essentiality of the SHMT reaction in generating folate-activated one-carbons from serine for dTMP synthesis in nuclei. 5-formyltetrahydrofolate pentaglutamate, a natural inhibitor of SHMT [11], did not inhibit nuclear dTMP biosynthesis but may not have been able to traverse the nuclear membrane. Disruption of nuclei by sonication eliminated all dTMP synthesis activity, indicating that cytoplasmic contamination was not responsible for the observed dTMP synthesis activity in nuclei, and suggesting that maintenance of nuclear architecture is essential for nuclear dTMP synthesis.

**Figure 3:**
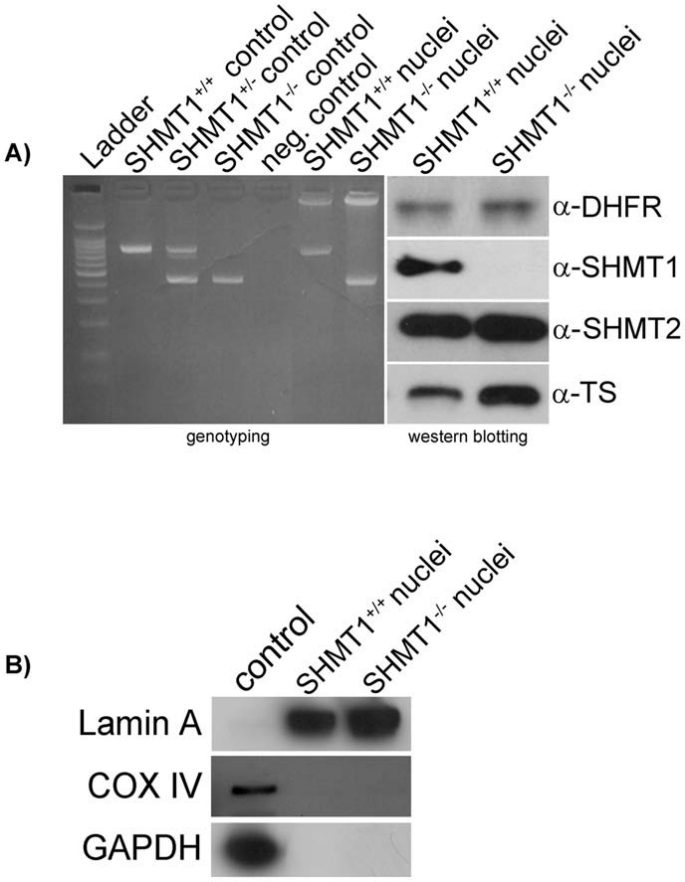
Identification of SHMT2 in purified nuclei. Panel A) PCR was used to confirm the genotype of nuclei isolated from *SHMT1^+/+^* and *SHMT1^−/−^* mice. *SHMT1^−/−^* mice were generated through deletion of exon 7 which encodes the PLP binding site. *SHMT1^+/+^* mice exhibit a 740-bp PCR product whereas the *SHMT1^−/−^* mice exhibit a 460-bp PCR product. Western blotting confirmed the presence of TYMS and DHFR in nuclei of both genotypes. SHMT1 was present in purified liver nuclei from *SHMT1^+/+^* mice, but absent in liver nuclei from *SHMT1^−/−^* mice. SHMT2 was present in nuclei from both genotypes. Panel B) Western blots confirm the purity of the isolated liver nuclei. The control lane represents purified cytosol from NIH/3T3 cells for both the Lamin A (nuclear marker) and GAPDH (cytoplasmic marker). The control for the COX IV immunoblot represents a purified mitochondrial fraction from NIH/3T3 cells. The absence of GAPDH and COX IV in nuclear extracts indicated that no cytosolic or mitochondrial contamination was present in the nuclear thymidylate biosynthetic assays.

Surprisingly, *intact* nuclei isolated from the liver of *Shmt1^−/−^* mice were capable of generating tritiated dTMP at approximately 25% of the level observed from nuclei isolated from *Shmt1^+/+^* mice (Figure 2). Aminomethylphosphonate inhibited dTMP synthesis in nuclei generated from *Shmt1^−/−^* mice, indicating that a second SHMT activity is present in nuclei which is not derived from *Shmt1*. PCR was used to confirm the genotype of the purified nuclei and immunoblotting was performed to verify that the nuclei lacked Shmt1 (Figure 3A). Furthermore, the nuclei were shown to be free of cytosolic and mitochondrial contamination (Figure 3B). DHFR and TYMS protein were observed in purified nuclei isolated from the livers of wild type and *Shmt1^−/−^* mice, and surprisingly, SHMT2 protein was also observed in nuclei isolated from the livers of wild type and *Shmt1^−/−^* mice (Figure 3A).

**Figure 4:**
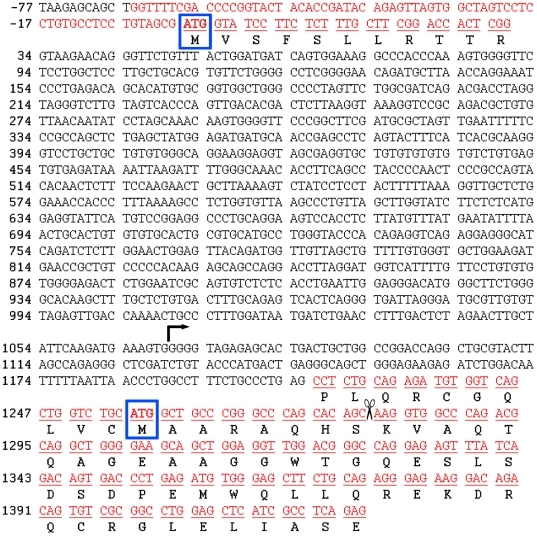
The mouse *Shmt2* encodes two transcripts and contains two translation initiation sites. The SHMT2 transcript is denoted by the color red. The first nucleotide of the translation initiation site contained in exon 1 is numbered as +1. The first translation initiation site in exon 1, which encodes for the mitochondrial leader sequence, is boxed in blue. The mitochondrial leader sequence is cleaved co-translationally in the mitochondria denoted by the scissors [2]. The second translation initiation site, present in exon 2, is boxed in blue. A transcription start site in intron 1 deduced from a mouse liver EST (AA793217) is denoted by the arrow and starts at −166 from the second ORF.

Previous evidence suggests that *Shmt2* contains two translation initiation sites. Expression of a *SHMT2* gene fragment lacking exon 1 in *glyA* CHO cells, which lack SHMT2 activity, rescued the glycine auxotrophy [4]. Exon 1 encodes the first translation initiation start site and most of the peptide sequence required for efficient import into mitochondria. *Shmt2* contains a potential second translation initiation codon within exon 2, and translation initiation from this site generated a protein capable of import into mitochondria, albeit at lower efficiency (Figure 4) [4]. To determine if two transcripts were generated from *Shmt2*, one containing exon 1 and one lacking exon 1, the mouse EST database was probed for *Shmt2* cDNA sequences that contained nucleotide sequence from the *Shmt2* intron 1/exon 2 boundary. An EST (AA793217) that lacked exon 1 but contained 166 nucleotides from the 3′ end of intron 1 at its 5′ end was identified. Similarly, the human EST database was probed and two *SHMT2* transcripts containing 131 nucleotides from the 3′ end of intron 1 were identified (DB184899 and DA597551). These data indicate that *SHMT2* encodes two transcripts and an alternative promoter within intron 1.

### Subcellular localization of SHMT2 gene products

**Figure 5:**
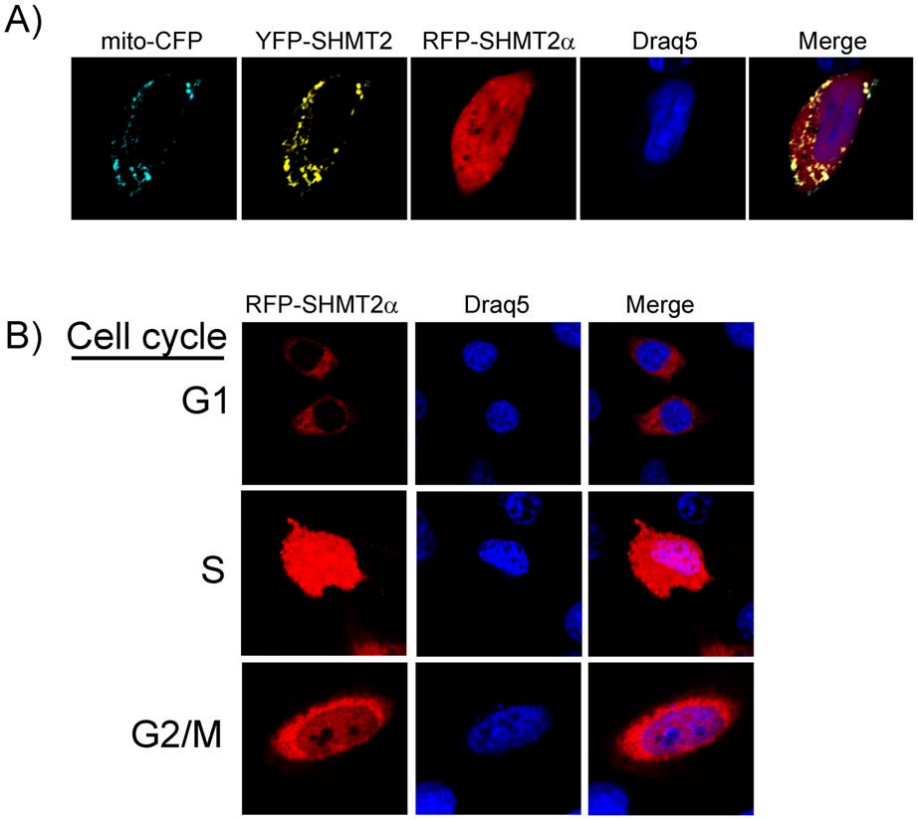
*SHMT2α* localizes to the cytoplasm and nucleus. HeLa cells were transfected with cDNAs encoding SHMT2-YFP, mito-CFP, and SHMT2α-RFP. A) The SHMT2-YFP fusion protein expressed in HeLa cells localizes to mitochondria. The mitochondrial marker, mito-CFP has a similar localization pattern as that of SHMT2. The SHMT2α-RFP fusion protein expressed in HeLa cells localizes to both the cytoplasm and the nucleus. B) SHMT2α-RFP localizes to the nucleus in a cell cycle dependent manner during S-phase and G2/M, whereas it is absent from the nucleus in G1 phase.

The cellular localization of the two SHMT2 isoforms was determined by expression of SHMT-yellow fluorescent protein (YFP) and red fluorescent protein (RFP) fusion proteins. Cyan fluorescent protein fused to a mitochondrial leader sequence (CFP-mito) at its amino terminus and Draq5 DNA binding dye were used as mitochondrial and nuclear markers respectively. Confocal microscopy revealed that the SHMT protein expressed from the *SHMT2* transcript containing exon 1 (referred to as SHMT2-YFP) and CFP-mito protein co-localized to mitochondria. However, SHMT protein expressed from the *SHMT2* transcript lacking exon 1 (referred to as SHMT2α-RFP) localized predominantly to the cytoplasm and nucleus (Figure 5A). The nuclear localization of the SHMT2α-RFP fusion protein exhibited similar cell cycle dependence as observed for SHMT1 [8]. The SHMT2α-RFP fusion protein localized exclusively to the cytoplasm in G1 phase whereas in S and G2/M phases it localized to both the cytoplasm and nucleus (Figure 5B).

### Both SHMT2 and SHMT2α rescue the glycine auxotrophy in GlyA Chinese Hamster Ovary cells

**Figure 6:**
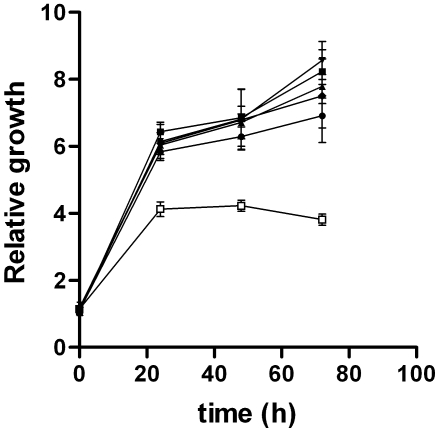
SHMT2 and SHMT2α rescue the glycine auxotrophy in CHO *glyA* cells. CHO *glyA* cells were transfected with cDNAs encoding human SHMT2-RFP, SHMT2α−RFP, and a RFP-empty vector control. Stable cells lines were selected for G418 resistance in the presence of 200 mM glycine. For growth assays, cells were cultured with and without glycine and MTT assays were used to quantify growth. Twelve independent lines were assayed per transfection and experiments were done in triplicate. All values are normalized to RFP-empty vector transfectants. There was no significant difference in growth among the cells transfected with SHMT2 and SHMT2α with or without glycine. RFP-empty vector transfectants with and without glycine are shown as a circle and open box respectively. SHMT2-RFP transfectants with and without glycine are shown as a closed box and triangle respectively. SHMT2α−RFP transfectants with and without glycine are shown as an inverted triangle and diamond respectively.

Previously, transfection of a human *SHMT2* gene fragment that lacked exon 1 into CHO *GlyA* cells, which lack SHMT2 activity in mitochondria and exhibit a glycine auxotrophy, resulted in very low levels of SHMT2 activity in mitochondria which was sufficient to rescue the glycine auxotrophy [4]. In this study, the ability of SHMT2α to rescue the glycine auxotrophy in *GlyA* cells was determined. Twelve to fifteen stable transfectants were selected for G418 resistance from *GlyA* cells electroporated with either an empty TagRFP-N plasmid, a plasmid expressing the cDNA encoding SHMT2, or a plasmid expressing the cDNA encoding SHMT2α. All plasmids were driven by the immediate early promoter of cytomegalovirus. Stable transfectants containing the RFP empty vector did not rescue glycine auxotrophy and no revertants were observed in the 12 colonies screened. However, the glycine auxotrophy was rescued in all selected cell lines expressing either the SHMT2-RFP or the SHMT2α-RFP fusion protein (Figure 6). This data confirms that SHMT2α localizes to mitochondria and can rescue mitochondrial one-carbon metabolism.

### Expression of SHMT2 and SHMT2α in mouse tissues

**Figure 7:**
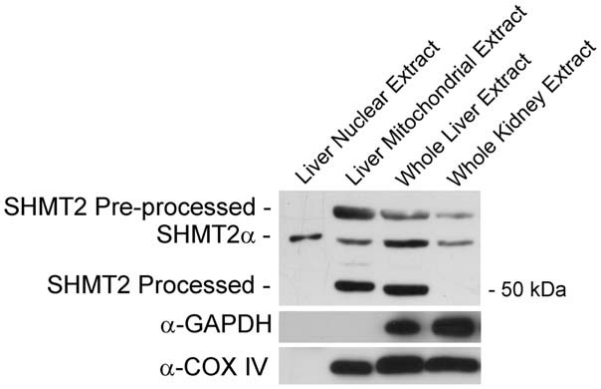
SHMT2 and SHMT2α are present in mouse tissues. Immunoblots using a sheep-anti-human SHMT2 antibody revealed the presence of three immunoreactive bands. Protein masses were determined by the migration distance and relative mobility of standards. Purified liver nuclei, whole liver and whole kidney extracts contained an immunoreactive band at ∼53 kDa, the predicted mass of SHMT2α. Purified liver mitochondria, whole liver and whole kidney extracts contained a band at ∼56 kDa, the predicted mass of the SHMT2 pre-processed protein. Purified liver mitochondria and whole liver, but not whole kidney extracts contained a band at ∼50 kDa, the predicted mass of the SHMT2 processed protein. The nuclear fraction was free of cytosolic and mitochondrial contamination as shown by the GAPDH and COX IV immunoblots.

The relative levels of each SHMT2 isoform was determined in mouse liver and kidney by western blot analyses (Figure 7). A single immunoreactive band that migrated at 53 kD was present in nuclear extracts isolated from mouse liver when probed with an antibody against SHMT2. This molecular mass corresponds to the predicted size of the SHMT2α protein expressed from the *Shmt2* transcript lacking exon 1. Purified liver mitochondrial extracts exhibited three immunoreactive bands. The upper band, which migrated with a molecular mass of approximately 56 kDa, is consistent with the predicted mass of the full length SHMT2 pre-processed protein. A lower band, which migrated with a molecular mass of approximately 50 kDa, corresponds to the processed SHMT2 protein lacking its leader sequence as present in mitochondria. These 3 bands were also observed in whole liver extract, and the blot indicates that the predominant SHMT2 isoform is the processed mitochondrial form. However, a substantial amount of the SHMT2α protein was also present in liver. In kidney, only the pre-processed SHMT2 and SHMT2α form of the enzyme were observed.

### A single nucleotide polymorphism in SHMT1 impairs nuclear localization

**Figure 8:**
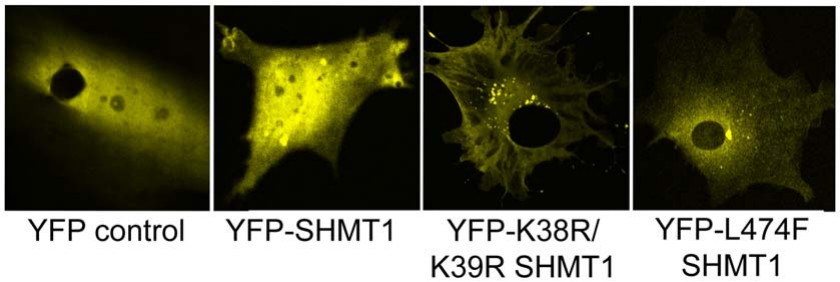
The L474F polymorphism in *SHMT1* impairs nuclear localization. *SHMT1^−/−^* MEF cells were transfected with cDNAs encoding the YFP-empty vector, YFP-SHMT1 wild-type, YFP-SHMT1-K38R/K39R, and YFP-SHMT1-L474F. S-phase blocked transfectants showed that YFP-SHMT1 is greatly increased in the nucleus in S-phase. Empty vector control and K38R/K39R mutation eliminated nuclear localization, and the L474F polymorphism inhibited nuclear localization.

Previously, we demonstrated that a common polymorphism in *SHMT1*, L474F impairs SUMO modification of the SHMT1 protein [8]. To determine if this polymorphism impairs nuclear localization and to explore the potential for the redundancy between SHMT1 and SHMT2α, MEFS derived from *Shmt1^−/−^* mice were electroporated with plasmids that express the wild-type human SHMT1-YFP fusion protein, the human L474F SHMT1-YFP fusion protein or a mutated K38R/K39R SHMT1-YFP fusion protein, which lacks a SUMO-modification site [8]. MEFs isolated from *Shmt1^−/−^* mice were used to eliminate the possibility that endogenous mouse SHMT1 protein could oligomerize with the human SHMT1 fusion proteins thereby allowing nuclear import. The SHMT1-YFP fusion protein localized to both the cytoplasm and nucleus in S-phase, whereas the K38R/K39R SHMT1-YFP fusion protein localized exclusively to the cytoplasm in S-phase (Figure 8). The L474F SHMT1-YFP fusion protein was found in both the cytoplasm and nucleus at S-phase, but in contrast to the SHMT1-YFP protein, its localization was primarily cytoplasmic. These data demonstrate that the *SHMT1* L474 polymorphism impairs nuclear localization. The potential for functional redundancy between SHMT1 and SHMT2α in nuclear folate metabolism may have been the permissive factor that allowed this mutation to expand in human populations.

## Discussion

The results from this study demonstrate directly the existence of nuclear thymidylate biosynthesis. Pardee and co-workers [10], [12], [13] first proposed the concept of nuclear nucleotide biosynthesis and put forward the concept of a nuclear multi-enzyme complex termed the replitase, which synthesized nucleotides at the replication fork during DNA synthesis. Both DHFR and TYMS activities were found in these purified complexes isolated from nuclei, but no direct evidence for nuclear nucleotide biosynthesis was reported. The concept of nuclear folate metabolism is also supported by early studies that demonstrated 10% of the total cellular hepatic folate was present within the nucleus [9]. Previous studies have shown that TYMS localizes to the nucleus at S-phase [8], [14], and recently SHMT1, DHFR, and TYMS proteins have all been shown to be SUMOylated, providing a mechanism by which all the enzymes required for *de novo* thymidylate biosynthesis can be localized to the nucleus [7], [8]. Recent evidence indicates that nuclear folate-dependent nucleotide biosynthesis is limited to thymidylate biosynthesis, as the multi-enzyme complex responsible for *de novo* purine biosynthesis, the purineosome, localizes to the cytoplasm [15].

In this study, we demonstrated that one-carbon units generated from serine are used for nuclear *de novo* thymidylate biosynthesis, and that the three enzymes that participate in *de novo* thymidylate pathway are present in nuclei. Compartmentalization of these enzymes to the nucleus accounts for previous isotope tracer studies that demonstrate SHMT1 preferentially shuttles one-carbon units towards thymidylate biosynthesis [6].

The data also demonstrate functional redundancy between *Shmt1* and *Shmt2* in nuclear folate metabolism. The observation that *Shmt2* (and human *SHMT2*) encodes a nuclear/cytoplasmic isozyme, SHMT2α, may account for the unexpected viability of *Shmt1^−/−^* mice, and potentially the emergence in human populations of the L474F *SHMT1* polymorphism, which impairs nuclear SHMT1 import. However, SHMT2α cannot fully replace SHMT1 function as *Shmt1^−/−^* mice exhibit elevated uracil content in DNA [16]. The S-phase and G2/M phase dependence of SHMT2α nuclear localization suggests that it is available for *de novo* thymidylate biosynthesis during DNA synthesis and repair as observed for SHMT1 [8]. The mechanism by which SHMT2α localizes to the nucleus is currently under investigation.

To our knowledge, *SHMT2* is the only gene identified that encodes proteins that can localize to the cytoplasm, nucleus and mitochondria. Previously, zebrafish SHMT2 protein was shown to localize exclusively to mitochondria [17]. The zebrafish *Shmt2* gene lacks the second translation initiation site in exon 2 and thus can only encode a mitochondrial isozyme. Mammalian *SHMT2* genes, with the exception of the lagomorph gene, encode a translation initiation codon in exon 2 and therefore likely encode the cytoplasmic/nuclear SHMT2α isozyme. These data also provide evidence for tissue-specific expression of the SHMT2 isoforms. The SHMT2/SHMT2α ratio differs between liver and kidney, with SHMT2 being the predominant form in liver, whereas only the SHMT2α and the SHMT2 unprocessed protein precursor were observed in kidney. Mitochondria are a primary source of one-carbon units for cytoplasmic one-carbon metabolism through the activity of SHMT2, and the SHMT2α protein lacks most of the mitochondrial targeting sequence and may not be imported into mitochondria efficiently [4]. Therefore, the lack of processed SHMT2 protein in kidney mitochondria may account for previous findings that glycine catabolism through the mitochondrial glycine cleavage system is a major source of one-carbon units in kidney [18]. The molecular mechanisms underlying the differential promoter usage in *SHMT2* that results in the synthesis of the SHMT2 isoforms is currently under investigation.

Compartmentation of SHMT2α and the other *de novo* thymidylate biosynthetic pathway enzymes in the nucleus may allow for folate dependent dTTP biosynthesis directly at the replication fork. It is likely that nuclear thymidylate synthesis requires the formation of an enzyme complex, as sonicated nuclei were not capable of dTMP biosynthesis. It has been shown that the processivity factor PCNA interacts with SHMT1 in both *C. elegans* and HeLa cDNA yeast two hybrids [8], [19]. Additional studies are required to determine why thymidylate biosynthesis, unlike purine nucleotide biosynthesis [15], occurs in the nucleus and if a thymidylate-specific replitase-like complex exists that enables the *de novo* dTMP synthesis pathway to function at the replication fork and prevent uracil misincorporation into DNA.

## Materials and Methods

### Nuclear thymidylate biosynthesis assay

The generation and characterization of *SHMT1^−/−^* mice has been described previously [16]. All study protocols were approved by the Institutional Animal Care and Use Committee of Cornell University and conform to the NIH Guide for the Care and Use of Laboratory Animals. Twelve livers were isolated from *Shmt1^+/+^* or *Shmt1^−/−^* mice on a 129SvEv background and placed immediately in cold phosphate buffered saline at 5°C. Liver extracts from six age-matched males and females were combined and used for each genotype group. Nuclei were prepared using an iodixanol gradient as previously described [20]. Purified nuclei were suspended in 500 µL of nuclear assay buffer containing 5 mM NADPH (Sigma), 100 mM β-mercaptoethanol, 25 mM HEPES, pH 7.5, 50 mM Sucrose, 5 mM MgCl_2_, 25 mM KCl, and 1 mM dUMP (Sigma) and quantified using a hemocytometer. 125 µL of assay buffer containing suspended nuclei were aliquoted into four 1.5 ml plastic tubes and 8 µCi of [2,3-^3^H]-L-Serine (Moravek Biochemicals) was added to each tube. The assay was conducted under 4 different experimental conditions: Experiment 1, nuclei that were lysed with sonication (Branson Sonifier 150) at 5°C using two 10 sec pulses at 10 watts separated by a 10 sec resting interval; experiment 2, intact nuclei; experiment 3, intact nuclei with 5-formyltetrahydrofolate pentaglutamate (Schirks Laboratory) added to at a final concentration of 200 µM; experiment 4, intact nuclei with aminomethylphosphonate added to a final concentration of 100 mM (Sigma). Reactions were incubated for 12 h at 37°C with shaking at 300 rpm. Nuclei were pelleted by centrifugation at 2000 rpm for 5 min and the supernatant was collected and analyzed for radiolabeled thymidylate by high performance liquid chromatography (HPLC). Sample preparation and HPLC was performed as previously described [21], [22]. Fractions were collected and tritium quantified with a scintillation counter. The retention times of [2,3-^3^H]-L-serine (9 min) and ^3^H-thymidine (17 min) (Moravek Biochemicals) were verified prior to separation of the reaction mixtures. All experiments were performed in duplicate.

### Genotyping and immunoblotting

After completion of the nuclear thymidylate biosynthesis reactions, pelleted nuclei were genotyped and analyzed by western blots. DNA was isolated using the DNeasy Blood and Tissue Kit (Qiagen) per manufacturer's protocol and genotyping as previously described to confirm the *Shmt1* genotype [16]. For western blots, nuclei were disrupted by boiling in SDS-PAGE loading buffer for 10 min and protein concentrations quantified by the Lowry-Bensadoun assay [23]. 20 µg of protein were loaded into each lane of a 10% SDS-PAGE gel[23]. Protein transfers, western blotting and DHFR and TYMS detection were performed as previously described [7]. SHMT1 and SHMT2 detection were completed as previously described [24], [25]. Equal loading and/or purity of nuclear fractions was confirmed through the detection of GAPDH using α-GAPDH (Novus Biologicals, 1∶20000 dilution), Lamin A using α-Lamin A (Santa Cruz Biotechnology, 1∶500 dilution), and COX IV using α-COX IV (Abcam, 1∶5000 dilution). For Lamin A detection, goat anti-rabbit IgG-horseradish peroxidase-conjugated secondary (Pierce) was used at a1∶20000 dilution. For COX IV and GAPDH detection, a 1∶10000 dilution of goat anti-mouse IgG-horseradish peroxidase-conjugated secondary (Pierce) was used.

The sizes of the SHMT2α isoforms were determined using the Precision Plus Protein All Blue (BIORAD) and Kaleidoscope Prestained Standard (BIORAD). Processed mitochondrial SHMT2 migrated with the Precision Plus Protein All Blue 50 kDa marker. To determine the size of the bands corresponding to preprocessed SHMT2 and SHMT2α proteins, a standard curve was generated by measuring the migration distances of the protein standards. These distances were plotted as a function of log_10_ of the molecular mass standards, and the molecular mass of preprocessed SHMT2 and SHMT2α were determined using the equation MM = −1.4D+137.9; *R^2^* = 0.98, where MM is molecular mass in kDa and D is migration distance in mm. The migration distance of the largest molecular mass band corresponding to preprocessed SHMT2 migrated 58 mm and SHMT2α migrated 61 mm, corresponding to approximately 56 kDa and 53 kDa respectively.

### Identification of Shmt2α expression

The nucleotide sequences of the human *SHMT2* and mouse *shmt2* intron 1/exon 2 boundaries were BLASTED against both the human and mouse EST databases to identify alternative *SHMT2* and *Shmt2* transcripts lacking sequence encoded by exon 1 but containing intron 1 sequence within the 5′-untranslated region. To identify *shmt2* protein products expressed from alternative *shmt2* transcripts, liver and kidney were isolated from *SHMT1^+/+^* mice on a C57Bl/6 background. The tissues were washed with phosphate-buffered saline and proteins solubilized using the Mammalian Protein Extraction Reagent (Pierce) containing 10 mM β-mercaptoethanol and a 1∶100 dilution of mammalian protease inhibitor (Sigma). Immunoblotting was performed as described above.

### Cell lines and culture

HeLa cells (CCL2) and NIH/3T3 cells (CRL-1658) were obtained from ATCC. Mouse embryonic fibroblasts were isolated and maintained as previously described [8]. *GlyA*, a CHO cell mutant lacking SHMT2 activity, was obtained from Dr. Larry Thompson, Lawrence Livermore Labs. All cells were cultured at 37°C in a 5% CO_2_ atmosphere. HeLa cells were maintained in minimal essential medium (α-MEM) (Hyclone) with 10% fetal bovine serum (Hyclone) and penicillin/streptomycin (Gibco). NIH/3T3 cells were cultured in DMEM (Gibco) supplemented with 10% fetal bovine serum and penicillin/streptomycin. *GlyA* cells were cultured in DMEM supplemented with 10% dialyzed and charcoal treated fetal bovine serum (*d*DMEM), 20 nM leucovorin (Sigma), 200 µM glycine (Sigma), and penicillin/streptomycin.

### Subcellular localization of SHMT2 and SHMT2α

The human *SHMT2* full length open reading frame was purchased from Open Biosystems. The cDNAs for the full length transcript (encoding SHMT2), and the transcript lacking exon 1 but containing 131 nucleotides of intron 1 in the 5′UTR (encoding SHMT2α) were amplified by PCR amplified and cloned into PhiYFP-N (Evrogen) and TagRFP-N (Evrogen) vectors respectively. The forward primer used to amplify the *SHMT2* transcript was 5′-ATATCTCGAGATGCTGTACTTCTCTTTGTT-3′. The forward primer to amplify the *SHMT2α* transcript was 5′-ATATCTCGAGGTATGGCCATTCGGGCTCAGCAC-3′. The underlined sequence signifies a *XhoI* (Promega) restriction site. The same reverse primer was used for both amplifications: 5′-ATATAAGCTTCTAATGCTCATCAAAACCAG-3′. The underlined sequence denotes a *HindIII* (Promega) restriction site. The PCR conditions for amplification of both the *SHMT2* and *SHMT2α* transcripts were as follows: 95°C for 45 s, 52°C for 45 s, and 72°C for 2 min. A vector containing a mitochondrial marker, pTagCFP-mito (Evrogen) was used for control transfections to identify mitochondria. Plasmids were transfected into HeLa cells using Lipofectamine 2000 (Invitrogen) per manufacturer's protocol. The DNA binding dye, Draq5 (Biostatus Limited) was used to visualize nuclei. For cell cycle analysis, HeLa cells were treated with 1 mM hydroxyurea, 30 µM Lovastatin or 60 ng/ml nocodazole to block at S, G_1_ and G2/M phase respectively as previously described [8]. Confocal fluorescence microscopy (Leica TCS SP2 system) was used to image all cells at the Cornell Microscope and Imaging Facility.

### Rescue of the glycine auxotrophy in GlyA cells

*GlyA* cells were cultured in *d*DMEM supplemented with 200 µM glycine and 20 nM leucovorin. TagRFP-N-*SHMT2* and TagRFP-N-*SHMT2α* and TagRFP-N vectors were linearized using *NheI* (Promega) and electroporated into *GlyA* cells as previously described [26]. Following electroporation, cells were plated on BD Primaria 100×20 mm culture dishes (BD Bioscience) containing 10 ml of *d*DMEM medium, supplemented with 20 nM leucovorin, 200 µM glycine and 200 µg/ml Geneticin (Gibco). 12–15 colonies from each transfection were isolated and plated in 6-well plates (Corning) containing 3 ml *d*DMEM medium supplemented with glycine, leucovorin, and Geneticin. Cell growth assays were completed in media with and without glycine as previously reported [27].

### Site-directed mutagenesis and subcellular localization of SHMT1

The *SHMT1* cDNA [28] was used as a template to generate SHMT1-YFP fusion proteins. The *SHMT1* cDNA was amplified for cloning into PhiYFP-N by PCR. The forward and reverse primers used were 5′- ATATCTCGAGATGACGATGCCAGTCAAC-3′ and 5′-ATATAAGCTTTTAGAAGTCAGGCAGGCC-3′ respectively. The PCR conditions were as follows: 95°C for 45 s, 55°C for 45 s, and 72°C for 2 min. The underlined regions depict *XhoI* and *HindIII* restriction sites respectively. Coding mutations K38R/K39R and L474F mutations were made as previously described [8]. PhiYFP-N vectors containing the *SHMT1* cDNA, the *SHMT1* cDNA containing a K38R/K39R mutation, and a *SHMT1* cDNA containing the L474F mutation were transfected into *Shmt1^−/−^* MEFs using electroporation protocols described above. Cells were blocked at S-phase by exposure to 1 mM hydroxyurea (Sigma) for 24 h. Confocal fluorescence microscopy (Leica TCS SP2 system) was used to image the SHMT1-YFP fusion proteins at the Cornell Microscope and Imaging Facility.
